# Garlic: a review of potential therapeutic effects

**Published:** 2014

**Authors:** Leyla Bayan, Peir Hossain Koulivand, Ali Gorji

**Affiliations:** 1 *Shefa Neuroscience Research Centre, Tehran, I. R. Iran *; 2 *Institut für Physiologie I, Klinik und Poliklinik für Neurochirurgie, Department of Neurology, Epilepsy Research Center, Westfälische Wilhelms-Universität Münster, Germany*

**Keywords:** *Cancer*, *Cardiovascular diseases*, *Herbal medicine*, *Traditional medicine*

## Abstract

Throughout history, many different cultures have recognized the potential use of garlic for prevention and treatment of different diseases. Recent studies support the effects of garlic and its extracts in a wide range of applications. These studies raised the possibility of revival of garlic therapeutic values in different diseases. Different compounds in garlic are thought to reduce the risk for cardiovascular diseases, have anti-tumor and anti-microbial effects, and show benefit on high blood glucose concentration. However, the exact mechanism of all ingredients and their long-term effects are not fully understood. Further studies are needed to elucidate the pathophysiological mechanisms of action of garlic as well as its efficacy and safety in treatment of various diseases.

## Introduction

Dietary factors play a key role in the development of various human diseases. Across cultures, there are many different dietary patterns which are believed to promote human health. Despite cultural differences, there are some shared characteristics of healthy dietary patterns. Perceiving plant foods as beneficial diet is advised by the folklore of many cultures over centuries. 

Garlic (*Allium sativum* L.) has acquired a reputation in different traditions as a prophylactic as well as therapeutic medicinal plant. Garlic has played important dietary and medicinal roles throughout the history.Some of the earliest references to this medicinal plant were found in Avesta, a collection of Zoroastrian holy writings that was probably compiled during the sixth century BC (Dannesteter, 2003 ▶). Garlic has also played as an important medicine to Sumerian and the ancient Egyptians. There is some evidence that during the earliest Olympics in Greece, garlic was fed to the athletes for increasing stamina (Lawson and Bauer, 1998 ▶). 

Ancient Chinese and Indian medicine recommended garlic to aid respiration and digestion and to treat leprosy and parasitic infestation (Rivlrn, 1998 ▶).In the medieval period, garlic was also played an important role in the treatment of different diseases. Avicenna (1988) ▶, in his well-known book, Al Qanoon Fil Tib (The Canon of Medicine), recommended garlic as a useful compound in treatment of arthritis, toothache, chronic cough, constipation, parasitic infestation, snake and insect bites, gynecologic diseases, as well as in infectious diseases (as antibiotic). With the onset of Renaissance, special attention was paid in Europe to the health benefits of garlic. Garlic has attracted particular attention of modern medicine because of widespread belief about its effects in maintaining good health. In some Western countries, the sale of garlic preparations ranks with those of leading prescription drugs. There is appreciable epidemiologic evidence that demonstrates therapeutic and preventive roles for garlic. Several experimental and clinical investigations suggest many favorable effects of garlic and its preparations. These effects have been largely attributed to *i*) reduction of risk factors for cardiovascular diseases, *ii*) reduction of cancer risk, *iii*) antioxidant effect, *iv*) antimicrobial effect, and *v*) enhancement of detoxification foreign compound and hepatoprotection (Colín-González, 2012 ▶; Aviello, 2009 ▶). In this review, a survey on current experimental as well as clinical state of knowledge about the preventive and therapeutic effects of garlic in different diseases is given. 

Garlic is a bulbous plant; grows up to 1.2 m in height. Garlic is easy to grow and can be grown in mild climates (Figure). There are different types or subspecies of garlic, most notably hardneck garlic and softneck garlic. Allicin (allyl 2-propenethiosulfinate or diallyl thiosulfinate) is the principal bioactive compound present in the aqueous extract of garlic or raw garlic homogenate. When garlic is chopped or crushed, allinase enzyme is activated and produce allicin from alliin (present in intact garlic). Other important compounds present in garlic homogenate are 1 -propenyl allyl thiosulfonate, allyl methyl thiosulfonate, (E,Z)-4,5,9-trithiadodeca- l,6,11-triene 9- oxide (ajoene), and y-L-glutamyl-S-alkyl- L-cysteine. The adenosine concentration increases several-fold as the homogenate is incubated at room temperature for several hours. 

Another widely studied garlic preparation is aged garlic extract. Sliced draw garlic stored in 15-20% ethanol for more than 1.5 year is refereed to aged garlic extract. This whole process is supposed to cause considerable loss of allicin and increased activity of certain newer compounds, such as S-allylcysteine, sallylmercaptocysteine, allixin, N-0 -(Ideoxy- D-fructos- 1 -yl)-L-arginine, and selenium which are stable and significantly antioxidant. Medicinally used, garlic oil is mostly prepared by steam-distillation process. Steam-distilled garlic oil consists of the diallyl, allylmethyl, and dimethyl mono to hexa sulfides (Lawson and Bauer, 1998 ▶). Botanically, *Allium sativum* is a member of the Lillaceae family, along with onions, chives, and shallots (Iciek et al., 2009 ▶; Lanzotti, 2006 ▶).

**Figure 1:**
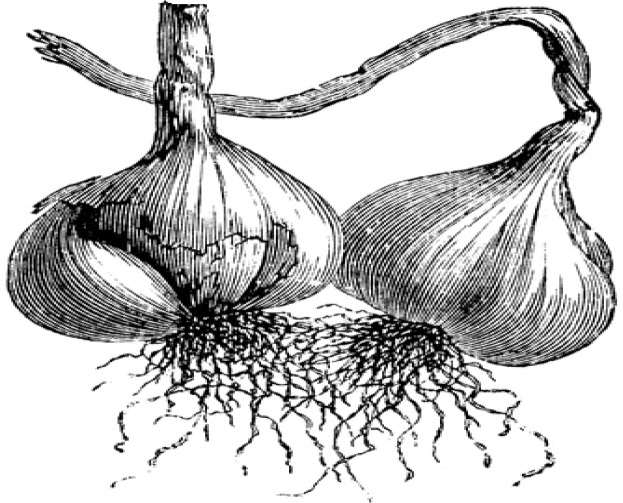
Garlic bulbs

**Effects of garlic on cardiovascular diseases**

Garlic and its preparations have been widely recognized as agents for prevention and treatment of cardiovascular diseases. The wealth of scientific literature supports the proposal that garlic consumption have significant effects on lowering blood pressure, prevention of atherosclerosis, reduction of serum cholesterol and triglyceride, inhibition of platelet aggregation, and increasing fibrinolytic activity (Chan et al., 2013 ▶). Both experimental and clinical studies on different garlic preparations demonstrate these favorable cardiovascular effects.

In *in vivo *animal experiments, intravenous administration of garlic extracts produced slight reductions in both systolic and diastolic pressures (Sial and Ahmed, 1982 ▶) and oral ingestion of garlic extract in hypertensive animals brought the blood pressure back to the normal level (Chandekar and Jain, 1973 ▶). Several clinical studies showed that garlic reduced blood pressure in more than 80% of patients suffering from high blood pressure (Auer et al., 1989 ▶; Konig and Scineider, 1986 ▶; Petkov, 1979 ▶; Omar, 2013 ▶; Stabler et al., 2012 ▶). In one trial, investigation on 47 hypertensive patients showed that garlic significantly decreased the mean systolic blood pressure by 12 mmHg and the mean supine diastolic blood pressure by 9 mmHg versus placebo. The authors stated that garlic was free from side effects and no serious complication was reported (Auer 1990 ▶). 

In another study, 200 mg of garlic powder was given three times daily, in addition to hydrochlorothiazide-triamterene baseline therapy, produced a mean reduction of systolic blood pressure by 10-11 mmHg and of diastolic blood pressure by 6-8 mmHg versus placebo (Kandziora 1988 ▶). However, these data are insufficient to determine if garlic provides a therapeutic advantage versus placebo in terms of reducing the risk of cardiovascular morbidity in patients diagnosed with hypertension (Stabler et al., 2012 ▶). 

It has been suggested that the mechanism of antihypertensive activity of garlic is due to its prostaglandin-like effects, which decrease peripheral vascular resistance (Rashid and Khan, 1985 ▶). Aged garlic extract was superior to placebo in lowering systolic blood pressure in patients suffering from uncontrolled hypertension. A dosage of 240-960 mg of aged garlic extract containing 0.6-2.4 of S-allylcysteine significantly lowered blood pressure by about 12 mmHg over 12 weeks (Ried et al., 2013a ▶).

Garlic administration in rats suffering from hypercholesterolemia, induced by a high-cholesterol diet, significantly reduced serum cholesterol, triglyceride, and LDL, but there was no effect on serum HDL (Kamanna and Chandrasekhara, 1982 ▶). In *in vitro* experiments, garlic administration suppressed LDL oxidation and increased HDL, which may be one of the protective mechanisms of the beneficial effects of garlic in cardiovascular health (Rahman and Lowe, 2006 ▶)   . Long term application of garlic and its preparations on experimental atherosclerosis induced by a high cholesterol diet, showed 50% reduction in atheromatous lesions, particularly in the aorta (Jain, 1977 ▶). Most of human studies on lipid lowering effects of garlic and garlic preparations described significant decrease in serum cholesterol and triglyceride (Gardner et al., 2001 ▶; Ziaei et al., 2001 ▶). A meta-analysis including 39 primary trials of the effect of 2 months administration of garlic preparations on total cholesterol, low-density lipoprotein cholesterol, high-density lipoprotein cholesterol, and triglycerides was performed (Ried et al., 2013b ▶). The results suggest garlic is effective in reduction of total serum cholesterol by 17±6 mg/dL and low-density lipoprotein cholesterol by 9 ± 6 mg/dL in subjects with elevated total cholesterol levels (>200 mg/dL). An 8% reduction in total serum cholesterol is of clinical relevance and is associated with a 38% reduction in risk of coronary events at 50 years of age. High-density lipoprotein cholesterol levels improved only slightly, and triglycerides were not influenced significantly. Garlic was highly tolerable in all trials and was associated with minimal side effects. 

This meta-analysis study concluded that garlic should be considered as an alternative option with a higher safety profile than conventional cholesterol-lowering medications in patients with slightly elevated cholesterol (Ried et al., 2013b ▶). However, a few studies using garlic powder, having low allicin yields, failed to show any lipid lowering effects (Lutomski, 1984 ▶; Luley et al., 1986 ▶). It has been suggested that different people might have different responses to garlic, thus garlic may be more beneficial for some specific groups (Zeng et al., 2013). 

Preventive effect of garlic on atherosclerosis has been attributed to its capacity to reduce lipid content in arterial membrane. Allicin, S-allyl cysteine, presented in aged garlic extract and diallyldi-sulfide, presented in garlic oil are the active compounds responsible for anti-atherosclerotic effect (Gebhardt and Beck, 1996 ▶; Yu-Yah and Liu, 2001 ▶). The plasma fibrinolytic activity in animals, which was decreased on cholesterol feeding, was considerably increased when this diet was supplemented with garlic (Mirhadi et al., 1993 ▶). 

Several human studies on plasma fibrinolytic activity have found that garlic increased fibrinolytic activity in healthy individuals as well as in acute myocardial infarction patients (Bordia et al., 1998 ▶). It was shown that pre-treatment with garlic significantly inhibited intracellular Ca^2+^ mobilization, thromboxane-A_2_ (a potent platelet aggregator) synthesis and protected against thrombocytopenia induced by collagen or arachidonate application in rabbits. 

These observations indicate that garlic may be beneficial in the prevention of thrombosis. Garlic has also been shown to inhibit platelet adhesion or aggregation in human investigations. It has been shown that the aged garlic extract inhibited the binding of ADP-activated platelets to immobilized fibrinogen. This suggested that aged garlic extract inhibited platelet aggregation via inhibition of the GPIIb/IIIa receptor and an increase in cAMP (Allison et al., 2012 ▶). Furthermore, it was reported that garlic decreases the risk of peripheral arterial occlusive diseases, plasma viscosity, and unstable angina and increases elastic property of blood vessels and capillary perfusion (Sumiyoshi and wargovich, 1990 ▶).

Seventy-eight patients with peripheral arterial occlusive disease were randomized to receive garlic or a placebo medication. The dose of garlic was 400 mg oral standardized garlic powder twice daily. Both men and women aged 40 to 75 years were enrolled in the study. After twelve weeks of treatment, pain-free walking distance increased similarly whether receiving garlic or placebo. Similarly there was no difference in the changes in blood pressure, heart rate, and pressure differences between the ankle and brachial pressures. No severe side effects were observed although more people taking garlic (28%) than placebo (12%) complained of a noticeable garlic smell. This indicates that any improvements in symptoms of peripheral arterial occlusive disease with garlic may require longer-term treatment and follow up than in this study (Jepson et al., 2000 ▶).

**Anti-tumor effect of garlic**

Many *in vitro* and *in vivo* studies have suggested possible cancer-preventive effects of garlic preparations and their respective constituents. Garlic has been found to contain a large number of potent bioactive compounds with anticancer properties, largely allylsulfide derivatives. Different garlic derivatives have been reported to modulate an increasing number of molecular mechanisms in carcinogenesis, such as DNA adduct formation, mutagenesis, scavenging of free radicals, cell proliferation and differentiation as well as angiogenesis. The growth rate of cancer cells is reduced by garlic, with cell cycle blockade that occurs in the G2/M phase (Capasso, 2013 ▶). In 1990, the U.S. National Cancer Institute initiated the Designer Food Program to determine which foods played an important role in cancer prevention (Dahanukar and Thatte, 1997 ▶). They concluded that garlic may be the most potent food having cancer preventive properties. Garlic has a variety of anti-tumor effects, including tumor cell growth inhibition and chemopreventive effects. In rodents, garlic and its constituents have been reported to inhibit the development of chemically induced tumors in the liver (Kweon et al., 2003 ▶), colon (Knowles and Milner, 2003 ▶), prostate (Hsing et al., 2002 ▶), bladder (Lau et al., 1986 ▶), mammary gland (Amagase and Milner, 1993 ▶), esophagus (Wargovich et al., 1988 ▶), lung (Sparnins et al., 1986 ▶), skin (Nishino et al., 1989 ▶), and stomach (Wattenberg et al., 1989 ▶) in both rodent and human studies. Diallyl trisulfide (DATS), an organosulfur compound isolated from garlic, has been shown anticancer activity both in *in vitro* and *in vivo* investigations. The cytotoxicity of DATS toward prostate epithelial cells reduced as opposed to PC-3 cancer cells (Borkowska, 2013 ▶). 

Possible anticarcinogenic mechanisms of garlic and its constituents may include the inhibition of carcinogen activation (Amagase and Milne, 1993 ▶), the enhancement of detoxification (Sumiyoshi and Wargovich, 1990 ▶), excretion (Tadi et al., 1991a ▶), and the protection of DNA from activated carcinogens (Tadi et al., 1991b ▶). Furthermore, DATS reduced tumor mass and number of mitotic cells within tumors. DATS reduced mitosis in tumors, decreased histone deacetylase activity, increased acetylation of H3 and H4, inhibited cell cycle progression, and decreased pro-tumor markers (survivin, Bcl-2, c-Myc, mTOR, EGFR, VEGF) (Wallace et al., 2013 ▶). Garlic components have been found to block covalent binding of carcinogens to DNA, enhance degradation of carcinogens, have anti-oxidative and free radical scavenging properties, and regulate cell proliferation, apoptosis, and immune responses. Ajoene, a garlic stable oil soluble sulfur rich compound and garlic-derived natural compound, have been shown to induce apoptosis in leukemic cells in addition to the other blood cells of leukemic patients. Ajoene induced apoptosis in human leukemic cells via stimulation of peroxide production, activation of caspase-3-like and caspase-8 activity. Garlic synergizes the effect of eicosapentaenoic acid, a breast cancer suppressor, and antagonizes the effect of linoleic acid, a breast cancer enhancer (Tsubura et al., 2011 ▶). 

Anti-proliferative activity of ajoene was demonstrated against a panel of human tumor cell lines (Li et al., 2002 ▶). Furthermore, allicin inhibits proliferation of human mammary endometrial and colon cancer cells. Growth inhibition is accompanied by an accumulation of the cells in *WIG1 *and G2lM phase of the cell cycle. Thus allicin is also responsible for the anti-proliferative effect of garlic derivatives. Diallyl sulfide and diallyl disulfide, inhibit arylamine N-acetyltransferase activity and 2-aminofluorene-DNA in human promyelocytic leukemia cells (Lin et al., 2002 ▶). Reduction of the risk of some malignancies by consumption of selenium-enriched plants, such as garlic was suggested (Finley, 2003 ▶). DATS inhibited cell growth of human melanoma A375 cells and basal cell carcinoma cells by enhancement of the levels of intracellular reactive oxygen species and DNA damage and by inducing endoplasmic reticulum stress and mitochondria-mediated apoptosis (Wang et al., 2012 ▶).

**Diabetes mellitus**

Although experimental studies demonstrated a clear hypoglycemic effect of garlic, the effect of garlic on human blood glucose is still controversial. Many studies showed that garlic can reduce blood glucose level in diabetic animals. Garlic was effective in reduction of blood glucose in streptozotocin- as well as alloxan-induced diabetes mellitus in rats and mice (Sheela et al., 1995 ▶; Ohaeri, 2001 ▶). Short term benefits of garlic on dyslipidemia in diabetic patients were shown (Ashraf et al., 2005 ▶). Garlic significantly reduced serum total cholesterol and LDL cholesterol and moderately raised HDL cholesterol as compared with placebo in diabetic patients (Ashraf et al., 2005 ▶). *S*-allyl cysteine, a bioactive component derived from garlic, restored erectile function in diabetic rats by preventing reactive oxygen species formation through modulation of NADPH oxidase subunit expression      (Yang et al., 2013 ▶).

Metformin and Garlic treatment in diabetic patients for 12 weeks reduced fasting blood glucose (FBG), but the percentage of change in FBG was more substantial with metformin supplemented with garlic than with metformin alone (Kumar et al., 2013 ▶). Chronic feeding of garlic extracts showed significant decrease in blood glucose level. However, some other studies showed no change of blood glucose level after that in human. Therefore, the role of garlic in diabetic patients needs to be further investigated (Banejee and Maulik, 2002 ▶). The beneficial effect of garlic on diabetes mellitus is mainly attributed to the presence of volatile sulfur compounds, such as alliin, allicin, diallyl disulfide, diallyl trisulfide, diallyl sulfide, S-allyl cysteine, ajoene, and allyl mercaptan. Garlic extracts have been reported to be effective in reducing insulin resistance (Padiya and Banerjee, 2013 ▶).

**Effect of garlic on chemically-induced hepatotoxicity**

Several studies showed that garlic can protect the liver cells from some toxic agents. Acetaminophen is a leading analgesic and antipyretic drug used in many countries. Overdose is known to cause hepatotoxicity and nephrotoxicity in humans and rodents. Although more than 90% of acetaminophen is converted into sulfate and glucouronide conjugates and excreted in the urine, a small portion is metabolized by different liver enzymes (Patten et al., 1993 ▶). This can arylate critical cell proteins and cause toxicity. It is demonstrated that garlic protects against acetaminophen-induced hepatotoxicity. Gentamycin also induces hepatic damage as revealed by elevation of liver damage marker enzymes (aspartate transaminase and alanine aminotransferase) and reduction in plasma albumin level. Dietary inclusion of garlic powder protects rats against gentamycin-induced hepatotoxicity, improves antioxidant status, and modulates oxidative stress (Ademiluyi et al., 2013 ▶). In addition, garlic attenuated hepatotoxicity effect of nitrate in rats. Garlic extract may reduce lipid peroxidation and enhance antioxidant defense system (El-Kott, 2012 ▶). 

**Anti-microbial effect of garlic**

Garlic has been used for centuries in various societies to combat infectious disease. Historically, it is believed that Louis Pasteur described the antibacterial effect of garlic in 1858 for the first time, although no reference is available. More recently, garlic has been proven to be effective against a plethora of gram-positive, gram-negative, and acid-fast bacteria. These include Salmonella, Escherichia coli (Adler and Beuchat, 2002 ▶), Pseudomonas, Proteus, Staphylococcus aureus (Cavallito, 1944 ▶), Escherichia coli, Salmonella (Johnson and Vaughn, 1969 ▶), Klebsiella (Jezowa and Rafinski, 1966 ▶), Micrococcus, Bacillus subtulis (Sharma et al., 1977 ▶), Clostridium (De Witt et al., 1979 ▶), Mycobacterium (Delaha and Garagusi, 1985 ▶), and Helicobacter (O’Gara et al., 2000 ▶). It has been documented that garlic exerts a differential inhibition between beneficial intestinal microflora and potentially harmful enterobacteria (Ress et al., 1993 ▶). 

The antibacterial activity of garlic is widely attributed to allicin. It is known that allicin has sulfhydryl modifying activity (Wills, 1956 ▶) and is capable of inhibiting sulfhydryl enzymes. Cysteine and glutathione counteract the thiolation activity of allicin. Garlic extract and allicin have been shown to exert bacteriostatic effects on some vancomycin-resistant enterococci. An inhibitory synergism was observed when used in combination with vancomycin (Jonkers et al, 1999 ▶). It is thought that allicin modifies the sulfhydryl groups on the enzymes of the TN1546 transposon, which encodes vancomycin resistance, enhancing susceptibility to vancomycin. 

The antibacterial effect of different concentrations of garlic extract against human dental plaque microbiota has been shown in *in vitro* study (Houshmand et al., 2013 ▶). The synergism between ciprofloxacin with garlic extract has been shown, but not between ampicillin and the garlic extracts (Zain al-abdeen et al., 2013 ▶). The cloves of garlic and rhizomes of ginger, extracted with 95% ethanol, suggested to have anti-bacterial activity against multi-drug clinical pathogens and can be used for prevention of drug resistant microbial diseases. Pseudomonas aeruginosa was the most sensitive germ to the mixture (Karuppiah and Rajaram, 2013 ▶). Garlic also suggested as a treatment for multi-drug resistant tuberculosis (Dini et al., 2011 ▶).

**Anti-protozoal properties**

Several studies have shown that the extract was effective against a host of protozoa including Candida albicans (Lemar et al., 2002 ▶), Scedosporium prolificans (Davis et al., 2003 ▶), tinea pedis (Ledezma et al., 2000 ▶), Opalina ranarum, Balantidium entozoon, Entamoeba histolytica, Trypanosomes, Leishmania, Leptomonas, and Crithidia (Reuter et al., 1966 ▶). 

Due to the occurrence of unpleasant side effects and increasing resistance to the synthetic pharmaceuticals, garlic was recommended for the treatment of giardiasis. Inhibitory activity of garlic on giardia was noted with crude extract at 25 pg/mlL and the lethal dosage was established as approximately 50 pg/mL. Encouraged by these results, a clinical trial was carried out on patients that had giardiasis (Soffar and Mokhtar, 1991 ▶). Garlic was established as an antigiardial, removing the symptoms from all patients within 24 h and completely removing any indication of giardiasis from the stool within 72 h at a dosage of 1 mg/mL twice daily aqueous extract or 0.6 mg/mL commercially prepared garlic capsules. No *in vitro* calculations were possible, as the workers could not culture the protozoa *in vitro*. It was suggested that allicin, ajoene, and organosulfides from garlic are effective antinrotozoals compounds.

**Antifungal properties**

Antifingal activity was first established in 1936 by Schmidt and Marquardt whilst working with epidermophyte cultures (Lemar et al., 2002 ▶). Many fungi are sensitive to garlic, including Candida (Yousuf, 2011 ▶), Torulopsis, Trichophyton, Cryptococcus (Fromtling and Bulmer, 1978), Aspergillus (Hitokoto et al., 1980 ▶), Trichosporon, and Rhodotorula (Tansey and Appleton, 1975 ▶). Garlic extracts have been shown to decrease the oxygen uptake (Szymona, 1952 ▶), reduce the growth of the organism, inhibit the synthesis of lipids, proteins, and nucleic acids (Adetumbi et al., 1986 ▶), and damage membranes (Ghannoum, 1988 ▶). 

A sample of pure allicin was shown to be antifungal. Removal of the allicin from the reaction by solvent extraction decreased the antifungal activity (Hughes and Lawson, 1991 ▶). Activity has also been observed with the garlic constituents, diallyl trisulfide, against cryptococcal meningitis (Cai, 1991 ▶), ajoene, and against Aspergillus (Yoshida et al., 1987 ▶). Thiol reduced the activity, suggesting the blocking of thiol oxidation by allicin. Inhibition of respiratory activity is thought to be due to inhibition of succinate dehydrogenase. The adhesion of Candida is also greatly reduced in the presence of garlic extract (Ghannoum, 1990 ▶). Again, this effect is diminished by the addition of thiol compounds. The addition of ajoene to some fungal growth mixtures, including Aspergillus niger, C. albicans, and Paracoccidiodes, has resulted in inhibition at concentrations lower than that experienced with allicin. Studies with aged garlic extract (with no allicin or allicin-derived constituents) showed no *in vitro* antifungal activity. However, when given to infected mice, the number of organisms that were seen was reduced by up to 80% (Tadi et al., 1991a ▶). 

It has been reported that garlic exhibited antifungal effects on two species, the air-borne pathogen Botrytis cinerea and Trichoderma harzianum (Lanzotti et al., 2012 ▶). Greater satisfaction with the use of garlic rather than nystatin was reported by the patients with denture stomatitis (Bakhshi et al., 2012 ▶).

**Antiviral properties**

In comparison with the antibacterial action of garlic, very little work has been done to investigate its antiviral properties. The few studies have reported that garlic extract showed *in vitro* activity against influenza A and B (Fenwick and Hanley, 1985 ▶), cytomegalovirus (Meng et al., 1993 ▶; Nai-Lan et al., 1993 ▶), rhinovirus, HIV, herpes simplex virus 1 (Tsai et al., 1985 ▶), herpes simplex virus 2 (Weber et al., 1992 ▶), viral pneumonia, and rotavirus. Allicin, diallyl trisulfide and ajoene have all been shown to be active (Hughes et al., 1989 ▶; Weber., 1992 ▶). 

In the case of HIV, it is thought that ajoene acts by inhibiting the integrin dependent processes (Tatarintsev et al., 1992 ▶). Allyl alcohol and diallyl disulfide have also proven effective against HIV-infected cells (Shoji et al., 1993 ▶). No activity has been observed with allicin or S-allyl cysteine. It appears that only allicin and allicin-derived substances are active. Taken together, the beneficial effects of garlic extract make it useful in medicine. There are insufficient clinical trials regarding the effects of garlic in preventing or treating the common cold. A single trial suggested that garlic may prevent occurrences of the common cold, but more studies are needed to validate this finding. This trial randomly assigned 146 participants to either a daily garlic supplement (with 180 mg of allicin content) or a placebo for 12 weeks. 

The investigation revealed 24 occurrences of the common cold in the garlic group compared with 65 in the placebo group, resulting in fewer days of illness in the garlic group compared with the placebo group. However, claims of effectiveness of garlic on common cold appear to rely largely on poor quality evidence (Lissiman et al., 2012 ▶). Many countries have used garlic extract for clinical treatments, but the untoward actions of garlic following long-term administration should be fully noted. Even though many studies on garlic and its derivatives have been performed, the exact biological mechanism of garlic extract still remains to be elucidated.

## Conclusion

A recent increase in the popularity of alternative medicine and natural products has renewed interest in garlic and their derivatives as potential natural remedies. This review may be useful to increase our knowledge of garlic therapeutic effects and improve our future experimental and clinical research plans. Although it is shown that garlic may have a significant clinical potential either in their own right or as adjuvant therapy in different disorders, however, due to some issues, such as methodological inadequacies, small sample sizes, lack of information regarding dose rationale, variation between efficacy and effectiveness trials, the absence of a placebo comparator, or lack of control groups more standard experiments and researches are needed to confirm the beneficial effect of garlic in various diseases. Future trials on the effect of garlic should include information on the dosage of active ingredients of standardized garlic preparations for better comparison of trials. It would also be interesting to explore the effect of different forms of garlic extract on standard drug therapy, especially when used as adjuvant therapy. 

Although garlic is believed to be a safe substance, long-term trials of reasonable duration would provide insights into the possible side-effects of different garlic extracts. The safety of garlic should be tested especially in pregnant or breastfeeding women as well as in young children (Budzynska et al., 2012; Dante et al., 2013). Long-term and large trials are also needed to evaluate the differences in mortality, serious adverse events, and morbidity of cancer and cardiovascular diseases after garlic therapy. 

## Conflict of interest

There is not any conflict of interest in this study.
